# RURAL DISPARITIES IN ELDERLY AMBULATORY CARE READMISSIONS: EVIDENCE FROM NATIONAL AMBULATORY MEDICAL CARE SURVEY

**DOI:** 10.1093/geroni/igae098.3180

**Published:** 2024-12-31

**Authors:** Sunny Kang, Nancy Miller, Ting Zhang

**Affiliations:** University of Baltimore, Baltimore, Maryland, United States; University of Maryland Baltimore County, Baltimore, Maryland, United States; University of Baltimore, Baltimore, Maryland, United States

## Abstract

**Objective:**

We examined factors associated with older patients’ readmission to ambulatory care within one week to two months’ discharge, and how this readmission pattern primarily varied by locality.

**Methodology:**

We analyzed 7,619 older patients (age 50 and older) from the 2016 National Ambulatory Medical Care Survey. Logistic regression analyses were utilized to examine the risk factors for ambulatory readmission. Findings: Compared to the residence in Metropolitan Statistical Areas, the residence in rural areas (or micropolitan areas) will increase the odds of readmissions (OR=1.61, 95%CI[1.30, 1.99]), after controlling for other variables. Being Black was not statistically significant in predicting the likelihood of readmission, while black patients using Medicaid as the primary payment experienced higher risks of readmission (OR=2.09, 95%CI[1.02, 4.32]). The higher facility percentage (>50%) of Medicare beneficiaries was associated with smaller odds of readmission (OR=0.65, 95%CI[0.58, 0.73]). Nursing home visiting within the prior week or seeing a physicians’ assistant each increased the odds of readmission, whereas seeing a mental healthcare provider decreased the risks for readmissions (OR=0.40, 95%CI[0.19, 0.83]). Patients diagnosed with cardiovascular disease and Type II diabetes were associated with smaller odds of readmission. Policy Implications: Residence in rural areas (or micropolitan statistical areas) or being black Medicaid beneficiaries predicted greater odds of readmission to ambulatory care among older patients. Rural patients experienced lower likelihood of staff responsiveness after adjusting for other factors. Further research should explore the basis of these disparities. Mental health diagnoses in depressive and psychosis disorders also called upon additional studies regarding special care needs.

